# Interplay Between *N*^6^-Methyladenosine (m^6^A) and Non-coding RNAs in Cell Development and Cancer

**DOI:** 10.3389/fcell.2019.00116

**Published:** 2019-06-28

**Authors:** Francesco Fazi, Alessandro Fatica

**Affiliations:** ^1^Department of Anatomical, Histological, Forensic and Orthopedic Sciences, Section of Histology and Medical Embryology, Sapienza University of Rome, Laboratory Affiliated to Istituto Pasteur Italia-Fondazione Cenci Bolognetti, Rome, Italy; ^2^Department of Biology and Biotechnology ‘Charles Darwin’, Sapienza University of Rome, Rome, Italy

**Keywords:** epitranscriptomics, m6A, RNA modifications, non-coding RNAs, microRNAs, lncRNAs, cell reprogramming, ESC development

## Abstract

RNA chemical modifications in coding and non-coding RNAs have been known for decades. They are generally installed by specific enzymes and, in some cases, can be read and erased by other specific proteins. The impact of RNA chemical modifications on gene expression regulation and the reversible nature of some of these modifications led to the birth of the word epitranscriptomics, in analogy with the changes that occur on DNA and histones. Among more than 100 different modifications identified so far, most of the epitranscriptomics studies focused on the *N*^6^-methyladenosine (m^6^A), which is the more abundant internal modification in protein coding RNAs. m^6^A can control several pathways of gene expression, including spicing, export, stability, and translation. In this review, we describe the interplay between m^6^A and non-coding RNAs, in particular microRNAs and lncRNAs, with examples of its role in gene expression regulation. Finally, we discuss its relevance in cell development and disease.

## Introduction

To date, more then 100 chemical modifications have been described in non-coding and protein coding RNAs (see The RNA Modification database^1^). The majority of them occur in transfer RNA (tRNA) and ribosomal RNA (rRNA), while a minority of them occur in messenger RNAs (mRNA) and long non-coding RNAs (lncRNAs). In all cases RNA modifications may play important role in RNA folding, stability and function; in view of the fact that, similarly to epigenetics, they can affect gene expression without changing the sequence of the RNA molecules, they are now referred to as “epitranscriptomics.” In metazoan, *N*^6^-methyladenosine (m^6^A) is the more abundant internal modification in mRNAs and lncRNAs and plays relevant roles in several steps of gene expression, including splicing, export, stability, and translation. Notably, m^6^A modification is present in all three different phylogenetic domains, Eukaya, Bacteria, and Archea (Carell et al., 2012), and is also present in viral RNAs (Lavi and Shatkin, 1975) where it has important regulatory functions (Meyer and Jaffrey, 2014). In analogy with DNA and histone modifications, m^6^A is a dynamic mark. It is installed by writers, removed by erasers and recognized by reader proteins. Despite its discovery in the early 1970s (Desrosiers et al., 1974; Perry and Kelley, 1974; Adams and Cory, 1975), the precise function of m^6^A residues in gene expression regulation remained elusive until recently with the development of high throughput methodologies for mapping of m^6^A residues in the whole transcriptome (Dominissini et al., 2012; Meyer et al., 2012; Linder et al., 2015). These methods used specific immunoprecipitation of m^6^A modified RNAs coupled to RNA sequencing. There are currently two different methodologies. The first one was developed by two independent groups (referred to as m^6^A-Seq or MeRIP-seq) and sequences immunoprecipitated m^6^A RNA fragment of about 200 nt, thus, does not allow for mapping of m^6^A residues at single-nucleotide resolution (Dominissini et al., 2012; Meyer et al., 2012). The second one (referred to as miCLIP), uses UV cross-linking to covalently bind the anti m^6^A antibody to modified RNAs, which induces mutation and truncation during reverse transcription, allowing for identification of single modified nucleotides within RNA species (Linder et al., 2015). m^6^A in mRNAs and lncRNAs can be installed by two independent complexes (Table 1): the heterodimeric complex of METTL3/METTL14 (methyltransferase-like protein 3 and 14), also referred to as MAC (m^6^A-METTL Complex), and the homodimeric complex of METTL16 (methyltransferase-like protein 16) (reviewed in Zhao et al., 2017; Lence et al., 2019). The MAC complex methylates adenosine during transcriptional elongation within the consensus motif RRACH (R = A/G; H = A/C/U), and is assisted in adenine selection by a multiprotein complex called MACOM (m^6^A-METTL-associated complex) composed of Wilms tumour 1-associated protein (WTAP), Vir-like m^6^A methyltransferase-associated (VIRMA), Cbl proto-oncogene like 1 (CBLL1, also known as Hakai), RNA-binding motif 15 (RBM15) or its paralog RBM15B, and zinc finger CCCH-type containing 13 (ZC3H13) proteins (Lence et al., 2019) (Table 1). The METTL3 is the catalytic component of the complex while METTL14 is required for RNA binding and stabilization. Recently, METTL14 was also found to interact with histone H3 trimethylation at Lys36 (H3K36me3), a marker for RNA polymerase II (RNA pol II) transcription elongation, thus ensuring modification of nascent RNAs in both intronic and exonic regions (Huang et al., 2019). However, m^6^A residues in mature mRNA molecules follow precise distribution and are enriched near the stop codon and untranslated regions. On the other hand, the METTL16 complex acts on a specific stem–loop structure of RNA containing the UACAGAGAA sequence. This complex acts on only a few percentages on methylated mRNAs and lncRNAs. However, between targeted RNAs, there is the human *S*-adenosylmethionine (SAM) synthetase MAT2A (Pendleton et al., 2017; Shima et al., 2017; Warda et al., 2017), which regulates cellular levels of the methyl donor SAM. Therein, METTL16 can regulate the activity of all cellular methyltransferases, including METTL3/MELL14. Moreover, it is also responsible for the methylation in the spliceosomal U6 small nuclear RNA (snRNA).

**TABLE 1:** Human m^6^A proteins.

**Protein**	**Function**	**References**
*Writers*		
METTL3	Installs m^6^A residues in mRNAs and lncRNAs	Liu et al.,2014
METTL14	Cooperates with METTL3 in m^6^A deposition	Liu et al.,2014
METTL16	Installs m^6^A in U6 snRNA and few mRNAs and lncRNAs	Pendleton et al., 2017; Warda et al.,2017
*Erasers*		
FTO	Remove m^6^A and m^6^Am from mRNA, and m^1^A from tRNA	Jia et al.,2011
ALKBH5	Remove m^6^A from mRNA	Zheng et al.,2013
*Regulators*		
WTAP	Regulates m^6^A installation by the METTL3/METTL14 complex	Ping et al.,2014
VIRMA	Regulates m^6^A installation by the METTL3/METTL14 complex	Knuckles et al., 2018; Yue et al.,2018
CBLL1	Regulates m^6^A installation by the METTL3/METTL14 complex	Wen et al., 2018; Yue et al.,2018
RBM15	Regulates m^6^A installation by the METTL3/METTL14 complex	Knuckles et al.,2018
ZC3H13	Regulates m^6^A installation by theMETTL3/METTL14 complex	Knuckles et al., 2018; Wen et al.,2018
*Direct readers*		
ABCF1	Stimulates cap-independent translation	Coots et al.,2017
eIF3	Stimulates cap-independent translation	Meyer et al.,2015
HNRNPA2B1	Stimulates microRNA processing	Alarcón et al.,2015a
IGF2BPs	Increase mRNA stability	Huang et al.,2018
YTHDC1	Stimulates splicing and mRNA export	Xiao et al., 2016; Roundtree et al.,2017
YTHDC2	Stimulates mRNA decay and translation	Hsu et al., 2017; Wojtas et al.,2017
YTHDF1	Stimulates translation	Wang et al.,2015
YTHDF2	Stimulates mRNA decay	Wang X. et al.,2014
YTHDF3	Stimulates mRNA decay and translation	Li et al., 2017; Shi et al.,2017
*Indirect readers*		
FMR1	Inhibits translation	Edupuganti et al.,2017
HNRNPC	Regulates splicing	Liu et al.,2015
*m^6^A repelled*		
ELAVL1	Increases mRNA stability	Wang X. et al.,2014
G3BPs	Increases mRNA stability	Edupuganti et al.,2017

m^6^A modification can be removed by two demethylase enzymes belonging to the AlkB family of the Fe(II) and α-ketoglutarate-dependent dioxygenases: ALKBH5 (alkB homolog 5) and FTO (fat-mass and obesity associated protein) (Zhao et al., 2017) (Table 1). The first one is specific for m^6^A removal while the second can also demethylate *N^6^, 2-O-dimethyladenosine* (m^6^Am), which is installed in mRNA if the first transcribed nucleotide is adenosine, and *N*^1^-methyladenosine (m^1^A) in tRNA (Wei et al., 2018).

Although m^6^A modification does not prevent Watson-Crick base pairing of A-U nucleotides, m^6^A residues can affect tertiary interactions involving Hoogsteen base pairs that use the N^6^ atom of A for H-bond (Meyer and Jaffrey, 2014). Therein, m^6^A residues within RNA molecules can produce local changing in the RNA structure that can alter RNA folding and affect the interaction with proteins and RNAs (Meyer and Jaffrey, 2014; Liu et al., 2015; Edupuganti et al., 2017). However, the function of m^6^A modifications in gene expression regulation is mainly mediated by m^6^A readers (reviewed in Patil et al., 2018) (Table 1). Proteins of the YT521-B homology (YTH) domain family were the first to be identified. In humans, there are five members: the nuclear YTHDC1, and the cytoplasmic YTHDC2, YTHDF1, YTHDF2, and YTHDF3. Mechanistically, YTHDC1 binding in the nucleus regulates alternative splicing and promotes RNA export while YTHDF2 binding stimulates mRNA decay and YTHDF1 binding promotes translation. The YTHDF3 reader can cooperate with both YTHDF1 and YTFDF2 on modified mRNA while in circular RNAs it can promote translation independently from other YTH proteins. Additional readers, lacking the YTH domains, have been also identified, including the translational regulators eIF3 and ABCF1, which positively regulate translation of modified mRNA and the insulin-like growth factor mRNA-binding proteins IGFBP-1, -2 and- 3, which enhance RNA stability and translation (Huang et al., 2018). Therein, once installed, m^6^A modifications can produce several different outputs on the regulation of specific RNAs which are not easily predictable.

Notably, m^6^A plays an important role in embryonic stem cells (ESCs) by controlling cell fate transition and deletion of METTL3 and METTL16 in mouse results in embryonic lethality, indicating essential function for m^6^A modification in the regulation of gene expression programs required for embryo development (Batista et al., 2014; Wang Y. et al., 2014; Geula et al., 2015; Bertero et al., 2018; Mendel et al., 2018). Moreover, m^6^A plays also important role in mouse adult brain, by regulating synaptic function and stress-induced responses (Engel et al., 2018; Koranda et al., 2018), and in the hematopoietic system, by controlling stem cell differentiation and homeostasis (Lv et al., 2018; Wang et al., 2018; Yao et al., 2018). Even if most of the m^6^A studies focused on its direct role on mRNA function, recent evidences showed that m^6^A can also regulate the synthesis and function of microRNAs and lncRNAs. In addition, microRNAs and lncRNAs can also influence the function of m^6^A modification in mRNAs. Here, we review the impact of m^6^A on the regulation of these non-coding RNAs and we discuss the interplay between m^6^A, microRNAs and lncRNAs in cell development and disease.

## Impact of Epitranscriptomics on MicroRNAs Biogenesis and Function

**FIGURE 1:**
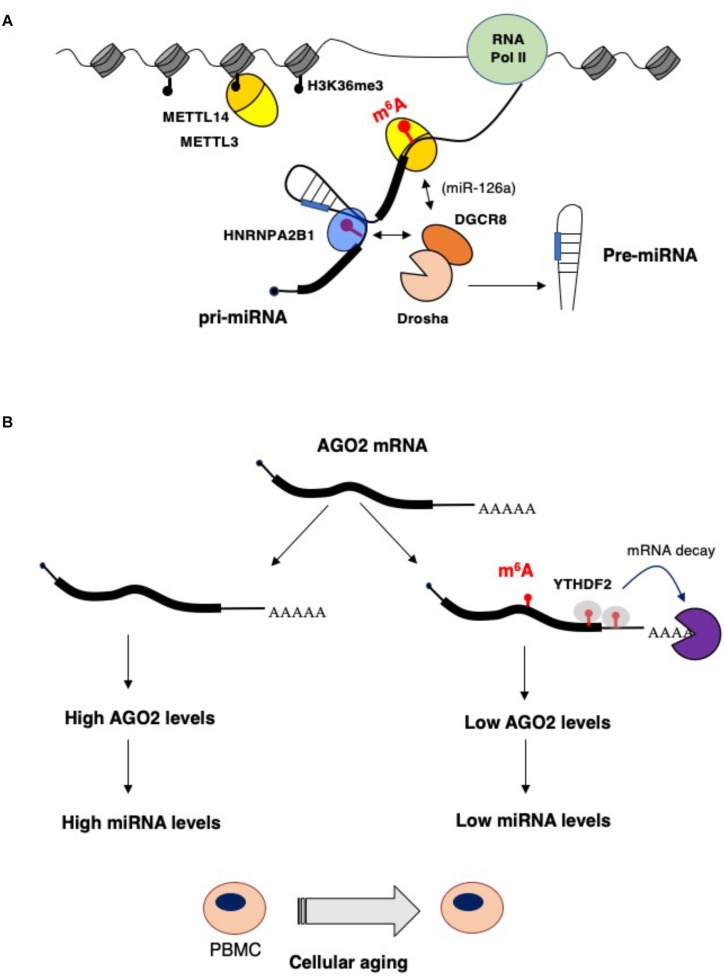
Impact of epitranscriptomics on microRNAs biogenesis and function. **(A)** m^6^A stimulates microRNA processing by recruiting the Drosha cofactor DGCR8 by the m^6^A reader HNBRPA2B1 (Alarcón et al., 2015a, b) or, in the case of the miR-126a, by direct interaction with METTL14 (Ma et al., 2017). **(B)** During aging of peripheral blood mononuclear cells (PBMCs), AGO2 and, eventually, miRNA levels are decreased by higher m^6^A modification of AGO2 mRNA. This results in enhanced mRNA decay that is very likely mediated by the YTHDF2 reader (Min et al., 2018).

MicroRNAs (miRNAs) are endogenously encoded short RNAs (∼21 nucleotides, nt) that are produced from long primary transcripts (pri-miRNA) transcribed by RNA Pol II (reviewed in Bartel, 2018). More than 50% of human miRNAs are encoded in introns of coding and non-coding pre-mRNAs and are connected to the expression of their host genes. The pri-miRNA is cleaved co-transcriptionally by a protein complex, named Microprocessor, containing the nuclear RNase III-type endonuclease *Drosha* and the DGCR8 (DiGeorge syndrome critical region gene 8) protein. The recognition of the pri-miRNA by the Microprocessor requires a stem–loop structure formed by the mature miRNA and single-stranded regions flanking the stem–loop. The cleavage by Drosha produces a stem–loop pre-miRNA of about 70 nt that is then recognized and exported to the cytoplasm by the transport receptor Exportin 5. In the cytoplasm, the pre-miRNA is cleaved by the RNase III-type endonuclease Dicer, releasing the miRNA duplex. One strand is then incorporated into the silencing complex containing one Argonaute (AGO) protein (in human AGO1, AGO2, AGO3, and AGO4) and the TNRC6 protein (also called GW182). The miRNA directs the Ago complex to its target mRNAs through perfect complementarity between sequences in the 3′ì untranslated region (3ì-UTR) and a stretch of 6 nucleotides (from nucleotides 2 to 7) in the 5′ region of the miRNA, also referred to as “seed.” The Ago proteins recruit TNRC6 protein, which stimulates mRNA deadenylation by interacting with deadenylase complexes and, consequently, produces mRNA destabilization and translational repression (Jonas and Izaurralde, 2015; Bartel, 2018). In addition, TNRC6 also recruits DDX6, a helicase that enhances both the decay and translational repression of target mRNAs (Jonas and Izaurralde, 2015; Bartel, 2018). m^6^A modification may affect microRNA synthesis and function at multiple levels (Figure 1A). A strong correlation between m^6^A residues in the 3′-UTR and miRNA-binding sites has been identified (Meyer et al., 2012). This has suggested the existence of a functional interaction between m^6^A modification and miRNAs targeting. In particular, the presence of m^6^A residues within the complementary region between the 3′-UTR and the miRNA seed might destabilize A-U pairing, thus decreasing duplex stability and affecting miRNA interaction. However, even if alterations in miRNA binding might contribute to some of the observed effects of m^6^A modification, the impact of m^6^A modifications within mRNAs on miRNA targeting is still not clear. On the other hand, m^6^A can also affect miRNA synthesis. m^6^A marks are deposited co-transcriptionally on a set of pri-miRNA molecules and are read by the HNRNPA2B1 proteins that, in turn, stimulate nuclear miRNA processing by recruiting the Microprocessor component DGCR8 (Alarcón et al., 2015a, b). Therein, alteration in m^6^A deposition may unbalance cellular miRNA levels. Moreover, upon acute temperature stress, the METTL3/METL14 complex can co-transcriptionally recruit the DGCR8 protein on stem–loop structures present in heat-shock genes, independently from the presence of an embedded precursor microRNA. Thus, promoting their subsequent nuclear degradation by the microprocessor complex (Knuckles et al., 2017). In view of the recent discovery of the association of METTL14 with chromatin during transcriptional elongation (Huang et al., 2019), we can speculate that the METTL3/METTL14 complex may contribute to the co-transcriptional recruitment of the Microprocessor complex on pri-miRNA transcripts. In addition, it has also been shown that, in hepatocellular carcinoma (HCC), METTL14 can directly recruit DCGR8 on the m^6^A modified pri-miRNA encoding for oncosuppressor miR-126a (Ma et al., 2017). In particular, low levels of METTL14 in HCC are associated with low levels of miR-126a and increased metastatic capacity (Ma et al., 2017). More importantly, ectopic expression of miR-126a in HCC cells ameliorated the metastatic phenotype induced by METTL14 downregulation (Ma et al., 2017).

Another example highlighting the role m^6^A modification on miRNAs processing has been recently reported for miR-25-3p. Zang and colleagues indeed described the impact of cigarette smoking on miR-25-3p maturation by m^6^A modification in pancreatic ductal adenocarcinoma (Zhang et al., 2019). In this manuscript, the authors described how the cigarette smoking induced the upregulation of METTL3 expression by affecting METTL3 promoter epigenetic regulation. This results in a METTL3-dependent modification of pri-miR-25-3p and an increase of miR-25-3p processing. The induction of miR-25-3p affects the expression of its target PH domain leucine-rich-repeats protein phosphatase 2 (PHLPP2), with an consequential impact on the AKT-p70S6K signaling pathway (Zhang et al., 2019). These results suggest that a METTL3-miR-25-3p-PHLPP2-AKT regulatory axis could be relevant for the transformation process induced by cigarette smoking in pancreatic tissue.

Interestingly, an additional mechanism leading to the modulation of m^6^A miRNAs modification is represented by the DDX3-dependent network. Indeed, DDX3, a member of the family of DEAD-box RNA helicases, has been shown to be able to interact with the RNA m^6^A demethylases, such as ALKBH5, resulting in m^6^A RNA demethylation. With specific regard to miRNAs, DDX3, thanks to its ability to also interact with AGO2 protein, may contribute to miRNAs demethylation. In summary, the functional contribution of DDX3 to the control of cell growth and proliferation may be at least in part mediated by its interaction with ALKBH5 and AGO2, relevant for the demethylation of mRNAs and miRNAs (Shah et al., 2017).

miRNA levels can also be controlled by m^6^A modification of AGO2 mRNA (Figure 1B). In a study performed on human peripheral blood mononuclear cells (PBMCs) from young and old donors, the AGO2 mRNA was found highly m^6^A methylated in young PBMCs and this correlated with a lower level of AGO2 mRNA in old PBMCs during aging (Min et al., 2018). AGO2 levels are important for both miRNA synthesis and function. Indeed, a lower level of AGO2 in old PBMCS resulted in an altered level of miRNA expression (Min et al., 2018), indicating that m^6^A modification on AGO2 mRNA contributes to cellular aging by regulating global miRNAs synthesis.

## Impact of Epitranscriptomics on LncRNA Regulation and Functions

LncRNAs are generally defined as transcripts longer than 200 nt without coding potential (reviewed in Fatica and Bozzoni, 2014). The human genome contains 16,193 genes encoding for lncRNAs (Gencode v30), which can produce more than 30,000 lncRNA transcripts. The majority of lncRNAs, but not all, share several features with coding mRNAs; they are 5′ capped, spliced and, in most of the cases, polyadenylated. Similar to mRNAs, lncRNAs are also m^6^A methylated and the levels of m^6^A residues strongly depend on the cell line, tissue type and growth condition (Meyer et al., 2012; Han et al., 2019; Xiao et al., 2019). In cell lines, the enrichment score of m^6^A peaks within mRNAs and lncRNAs is very similar (Han et al., 2019). However, it has been shown recently that in human fetal tissues, a lower proportion of lncRNA is m^6^A modified compared to mRNA (Xiao et al., 2019). In contrast to mRNAs, m^6^A residues in lncRNAs are distributed along the whole body of the transcript and are more present in lncRNAs that undergo alternative splicing (Xiao et al., 2019). Thus, this indicates a possible function for m^6^A modification in regulation of lncRNA isoforms. Many lncRNAs are retained and function in the nucleus. Nuclear lncRNA may regulate gene expression by several mechanisms, such as modulating the activity of regulatory protein complexes, regulating chromosomal conformations and, more generally, nuclear organization (reviewed in Engreitz et al., 2016). In particular, different lncRNAs regulate gene expression by guiding regulatory complex to specific gene *loci*. This is generally achieved by lncRNA interaction with chromatin associated proteins, local chromosomal architecture or by forming an RNA-DNA triple helix (Engreitz et al., 2016; Li et al., 2016). LncRNA local structure and interaction with specific proteins plays an important role in lncRNA function. Therein, m^6^A modification might regulate lncRNA function by providing binding sites for m^6^A reader proteins or, alternatively, might regulate local RNA structure to allow access for specific RNA-binding proteins to nearby m^6^A residues. Furthermore, m^6^A modification may also influence RNA–DNA triple helix formation, in which a lncRNA binds with sequence specificity through Hoogsteen base pairs in the major groove of a Watson–Crick base-paired DNA duplex. Therein, m^6^A modification can potentially affect lncRNA interaction with specific DNA *loci*.

Metastasis-associated lung adenocarcinoma transcript 1 (MALAT1), also known as nuclear-enriched abundant transcript 2 (NEAT2), is a highly expressed nuclear lncRNA, frequently upregulated in cancer, that contains several m^6^A modifications (Dominissini et al., 2012). Even if MALAT1 is transcribed by RNA pol II, it lacks a canonical poly-A tail. The high stability observed for MALAT1 transcript is ensured by a triple-helix at its 3′-end that specifically binds METTL16 (Brown et al., 2014, 2016). MALAT1 accumulates in the nuclear speckels, which are nuclear domains enriched in splicing factors, and associates with different splicing regulators such as the serine/arginine-rich (SR) proteins and the protein heterogeneous nuclear ribonucleoprotein C (Tripathi et al., 2010; Zhou et al., 2016). In cell lines, MALAT1 silencing alters the alternative splicing of specific pre-mRNAs (Tripathi et al., 2010). However, MALAT1 knock-out in mouse has no effect on the alternative splicing nor on the formation of nuclear speckles (Nakagawa et al., 2012; Zhang et al., 2012). A further study showed that MALAT1 can also bind to the Polycomb 2 protein (Pc2), a component of the Polycomb Repressive Complex -1 (PRC1), and that it can act as scaffold in distinct subnuclear compartments required for coordinated regulation of gene transcription (Yang et al., 2011). Notably, m^6^A modifications identified in MALAT1 can alter the accessibility of the RNA motif to which proteins bind, through changing its local structure (Liu et al., 2015; Spitale et al., 2015; Zhou et al., 2016), a mechanism known as “m^6^A switch” (Liu et al., 2015). Therein, m^6^A might affect the function of MALAT1 in splicing and transcription by regulating RNA-protein interactions.

MALAT1 can also interact with microRNAs and act as a competing endogenous RNA (ceRNA), thus affecting microRNA binding to target mRNAs, in different cell types (Leucci et al., 2013; Han et al., 2015; Hirata et al., 2015; Xiao et al., 2015). Even in some cases, the interaction of MALAT1 with microRNAs has been reported to occur in the nucleus (Leucci et al., 2013), whereas in other cases, a specific translocation of MALAT1 in the cytoplasm is required (Han et al., 2015). However, the mechanism responsible for its translocation is still not known. Notably, m^6^A modification is directly involved in RNA nuclear export (Zheng et al., 2013; Roundtree et al., 2017; Lesbirel et al., 2018). Therein, the level of m^6^A modifications within MALAT1 might directly control its cellular localization and, eventually, its ceRNA activity. Alternatively, m^6^A modifications might directly regulate the RNA-RNA interactions between MALAT1 and targeted microRNAs, as recently reported for another ceRNA (Yang et al., 2018).

The X-inactive specific transcript (Xist), an important regulator of X-chromosome inactivation in mammals, is another highly m^6^A methylated lncRNA. An initial shRNA screening performed in mESCs identified three components of the MACOM complex, WTAP, VIRMA (also known as KIAA1429) and RBM15 proteins, which are all regulators of Xist activity (Moindrot et al., 2015). Moreover, knockdown of RBM15 and WTAP greatly impaired Xist mediated epigenetic silencing (Moindrot et al., 2015). In addition, WTAP protein was also identified as a stable interactor of Xist RNA (Chu et al., 2015). More recently, it has been confirmed that m^6^A modification is strictly required for Xist-mediated transcriptional repression and that knockdown of the METTL3 writer inhibits X chromosome silencing (Patil et al., 2016). The RBM15 protein was identified as the MACOM components that interacts with and guides the METTL3/METTL14 complex for the formation of the 78 m^6^A present in Xist RNA (Patil et al., 2016). Furthermore, it was also shown that the YTHDC1 reader recognized the m^6^A marks in mESCs and is required for Xist activity even if the mechanism has not yet been clarified (Patil et al., 2016). Notably, tethering of YTHDC1 on Xist in the absence of m^6^A residues is sufficient for its repressive function, indicating that m^6^A *per se* is not required for Xist activity. YTHDC1 is the only nuclear reader of the YTH family and is usually involved in the regulation of pre-mRNA spicing through recruiting splicing factors (Xiao et al., 2016). Similarly, YTHDC1 binding on Xist might function by bridging *cis*- acting regulatory elements on XIST RNA with *trans*-acting proteins required for transcriptional silencing.

Recently, it has also been found that the enhancer RNAs (eRNAs), non-coding transcripts produced from enhancer regions that act as regulators of transcription, are highly m^6^A modified (Xiao et al., 2019). This has suggested that m^6^A modification might contribute to the enhancer function of eRNAs during transcription.

In the cytoplasm, lncRNAs may regulate mRNA stability and translation by recruiting regulatory proteins to interacting mRNAs or by acting as ceRNAs (reviewed Fatica and Bozzoni, 2014). Therein, m^6^A residues might affect cytoplasmic lncRNA function with the same mechanisms described above. A recently identified cytoplasmic lncRNA whose function is regulated by m^6^A is lincRNA 1281 (linc1281) (Yang et al., 2018). Linc1281 is required for mESC differentiation and acts as a ceRNA by sequestering miRNAs of the let-7 family (Yang et al., 2018). Notably, linc1281 contains different m^6^A marks in its 3′-end region that are required for the binding of let-7 (Yang et al., 2018). It has been proposed that the presence of m^6^A in linc1281 can act as m^6^A-switch for specific RNA binding proteins, which will eventually regulate the interaction with let-7. However, the identity of such proteins has not yet been discovered. A similar mechanism has been already proposed for the binding of HuR (ELAVL1) protein and miRNAs to mRNAs encoding developmental regulators in mESCs (see below).

In view of the fact that many lncRNAs can be m^6^A modified and that m^6^A can affect their expression levels and functions, it very likely that other examples of lncRNAs regulated by m^6^A modification will follow soon.

## Interplay Between Non-Coding RNAs and m^6^A Effector Proteins

**FIGURE 2:**
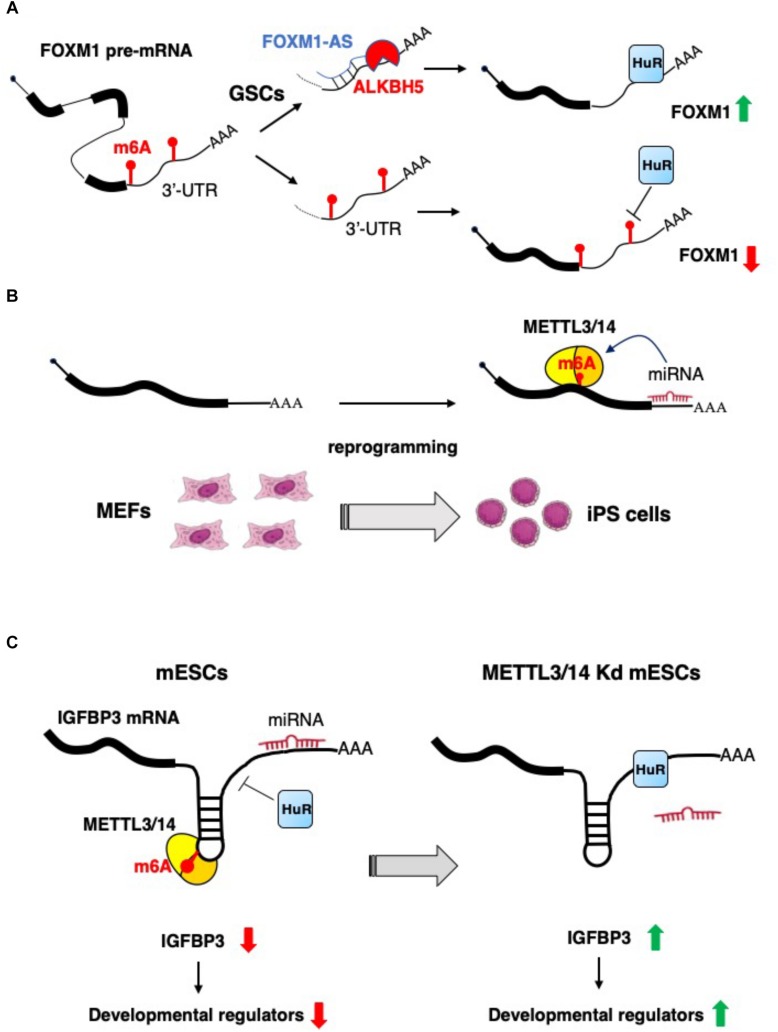
Examples of the interplay between non-coding RNAs and epitranscriptomics. **(A)** In glioblastoma stem-like cells (GSCs) the expression of FOXM1 is increased by the concomitant expression of the antisense transcript FOXM1-AS, which, in turn, promote m^6^A demethylation by recruiting ALKBH5 (Zhang et al., 2017). **(B)** m^6^A RNA methylation is positively regulated by microRNAs, which recruit METTL3 on specific mRNA and promotes reprogramming to pluripotency (Chen et al., 2015). **(C)** m^6^A modification decreases the IGFBP3 mRNA levels by inhibiting the binding of HuR and promoting the interaction with microRNAs. IGFBP3 protein positively regulates the stability of different developmental regulators. This mechanism ensures low level of IGFBP3 in mESCs (Wang Y. et al., 2014).

As highlighted above, m^6^A is a relevant modification for non-coding RNA biogenesis and functional activity. However, it has been also reported that lncRNAs may control the function of the epitranscriptomics machinery. For example, the expression of the nuclear lncRNA FOXM1-AS allows the interaction between the FOXM1 nascent RNA and the m^6^A demethylase ALKBH5, that results in the demethylation of FOXM1 transcripts. This promotes the binding of HuR protein (also known as ELAVL1) with FOXM1 pre-mRNA, resulting in an elevated expression of FOXM1. Interestingly, it has also been shown that, in glioblastoma stem-like cells (GSCs), the m^6^A demethylase ALKBH5 is highly expressed, and the depletion of ALKBH5 and FOXM1-AS disrupts GSC tumorigenesis through the reduction FOXM1 expression (Zhang et al., 2017) (Figure 2A).

Recently, an additional function of non-coding RNAs in the control of m^6^A modification has emerged. In particular, it has been reported that the expression of epitranscriptomics machinery components may be controlled by miRNAs through the targeting of their corresponding mRNAs. Here, we include some representative examples of such modulation.

miR-145, broadly reported as a tumor suppressor miRNA, was shown to control the expression of the YTHDF2 reader. YTHDF2 is involved in the deadenylation and decay of m^6^A-containing RNAs through a direct interaction and recruitment of the CCR4-NOT deadenylase complex (Du et al., 2016).

Specifically, in liver cancer cells, miR-145 downregulates YTHDF2 mRNA expression with an increase on the overall levels of mRNAs containing m^6^A residues, as evaluated by dot-blot and immunofluorescence analyses with anti m^6^A antibodies, which do not allow m^6^A mapping in specific RNA species (Yang et al., 2017). Interestingly, this increase is inhibited by YTHDF2 overexpression, supporting the central role of this protein in this regulation (Yang et al., 2017). Accordingly, miR-145 expression levels are negatively correlated with those of YTHDF2 in HCC tissues. Functionally, miR-145 is able to suppresses the proliferation of HCC cells through the modulation of m^6^A-modified mRNA levels by targeting the 3′-UTR of YTHDF2 mRNA.

Another example is represented by miR-33a. In non-small-cell lung carcinoma (NSCLC) cells, it was recently described that miR-33a, by targeting the 3′-UTR of METTL3 mRNA, reduces the expression of METTL3 at both mRNA and protein levels and, eventually, global m^6^A mRNA methylation, with a functional reduction of cellular proliferation and anchorage-independent growth (Du et al., 2017).

Recently, it has been reported that the oncogenic properties of glioblastoma stem cells (GSCs) in terms of proliferation, migration, and invasion could be influenced by a novel miR-29a/QKI-6/WTAP molecular axis (Xi et al., 2017). Of note, the overexpression of miR-29a inhibits WTAP expression and the activation of the ERK and PI3K/AKT pathways by downregulating QKI-6 expression and impairing the oncogenic abilities of GSCs (Xi et al., 2017). The impact of this regulatory network on m^6^A levels have not been addressed in this study. However, considering the relevance of WTAP in regulating the methylation activity of the METTL3-METTL14 complex (Ping et al., 2014), it might be speculated that miR-29a, besides regulating DNA methylation during cell reprograming by targeting DNA-methyl-transferases (DNMTs) (Hysolli et al., 2016), could also have a crucial role in the modulation of RNA m^6^A methylation during neoplastic transformation processes.

Lately, a novel role of miRNAs in the regulation of m^6^A modification has emerged. Specifically, an enrichment of seed sequences for miRNAs has been observed in transcripts presenting m^6^A residues using next generation sequencing (NGS) approaches (Chen et al., 2015). This study evaluated the contribution of miRNAs to the *ab initio* induction of m^6^A methylation by depletion or overexpression of Dicer to modulate the overall miRNAs activity. In particular, Dicer expression favors the induction of m^6^A methylation on target mRNAs, highlighting the tight connection between miRNAs activity and m^6^A modification. Mechanistically, Dicer promotes the localization of METTL3 in nuclear speckles, enhancing its interaction with the transcript subjected to m^6^A modification. In the same study, by using m^6^A-Seq, the authors identified m^6^A levels specific to different degrees of pluripotency by analyzing various experimental models, such as ESCs, induced pluripotent stem cells (iPSCs), neural stem cells (NSCs), and testicular sertoli cells (SCs). They revealed the existence of both cell-common and cell-specific modified transcripts associated with biological processes such as stem cell maintenance and cell differentiation. Moreover, to explore the contribution of m^6^A modification in cell reprograming, the authors overexpressed human METTL3 into mouse embryonic fibroblasts (MEFs), expressing the reprograming factors Oct4, Sox2, Klf4, and c-Myc, and evidenced that the reprograming efficiency of MEFs was significantly improved by the increase METTL3-dependent m^6^A levels (Chen et al., 2015) (Figure 2B).

Of note, a role for m^6^A as a signal for miRNA-dependent degradation of transcripts encoding developmental regulators in mESCs has also recently emerged. Indeed, during mESCs normal development, the METTL3/METTL14-dependent m^6^A methylation of transcripts encoding developmental regulators blocks HuR protein binding and results in miRNAs-mediated transcript destabilization (Wang Y. et al., 2014). The loss of m^6^A modification, in METTL3 and METTL14 knocked-down cells, allows for HuR-mRNA interaction and reduction of miRNA functional activity, improving transcripts stability and promoting loss of the mESC ground state (Wang Y. et al., 2014) (Figure 2C).

On the contrary, it has also been shown that the presence of m^6^A modification may serve as a protective signal, which inhibits mRNA degradation through the binding of the IGF2BPs readers. In the case of SRF transcript, m^6^A modification allows interaction with IGF2BP inhibiting miRNA-mediated decay in cancer cells (Müller et al., 2019). SRF induction is associated with tumor cell phenotype and poor prognosis (Muller et al., 2019). In addition, IGF2BP1 can also stabilize different oncogenic mRNAs by inhibiting general mRNA degradation, as reported for MYC mRNA (Huang et al., 2018).

## Conclusion

We are witnessing an impressive increase in the number of studies elucidating the role of m^6^A modification in cell development and cancer. However, the majority of these studies mainly focused on the impact of m^6^A marks on coding RNAs, while an important contribution of ncRNA molecules is emerging, such as lncRNAs and microRNAs, on the function of the epitranscriptome. Moreover, lncRNA and miRNA function can be itself regulated by m^6^A. Nevertheless, different questions still need to be answered. In particular, mapping of m^6^A modification in mRNAs and lncRNAs showed a different distribution of m^6^A marks within these two types of RNA pol II transcripts. However, it is still not known how this is achieved and, above all, if specific regulatory factors act differentially in controlling the m^6^A methylases activity on mRNA and lncRNA molecules. Moreover, these studies have mainly used polyA^+^ RNA for m^6^A mapping, therein excluding many regulatory ncRNAs missing a polyA tail. Other important remaining questions in the field will be to determine if small ncRNAs such as miRNAs or the recently functionally characterized Y RNAs contain m^6^A residues and if these are relevant for their regulatory function in normal and pathological conditions. Furthermore, another important issue concerns the impact of the interplay between m^6^A modification and non-coding RNA molecules on cell fate determination and development, and which are relevant for these biological processes. In this review, we have described some examples, but the general impact of this emerging molecular network will be clarified after extensive further investigation.

## Author Contributions

All authors listed have made a substantial, direct and intellectual contribution to the work, and approved it for publication.

## Conflict of Interest Statement

The authors declare that the research was conducted in the absence of any commercial or financial relationships that could be construed as a potential conflict of interest.

## Footnotes

1 https://mods.rna.albany.edu/mods/
